# HCMV US2 co-opts TRC8 to degrade the endoplasmic reticulum-resident protein LMAN2L

**DOI:** 10.1099/jgv.0.001980

**Published:** 2024-04-30

**Authors:** Leah M. Hunter, Joanne Kite, Alice Fletcher-Etherington, Katie Nightingale, Luis Nobre, Robin Antrobus, Ceri A. Fielding, Richard J. Stanton, Michael P. Weekes

**Affiliations:** 1 Cambridge Institute for Medical Research, University of Cambridge, Hills Road, Cambridge CB2 0XY, UK; 2 Department of Medicine, University of Cambridge, Hills Road, Cambridge, CB2 2QQ, UK; 3 Cardiff University School of Medicine, Division of Infection and Immunity, Henry Wellcome Building, Heath Park, Cardiff CF14 4XN, UK

**Keywords:** cytomegalovirus, protein degradation, protein trafficking, plasma membrane, proteomics

## Abstract

The human cytomegalovirus (HCMV) pUS2 glycoprotein exploits the host’s endoplasmic reticulum (ER)-associated degradation (ERAD) pathway to degrade major histocompatibility complex class I (MHC-I) and prevent antigen presentation. Beyond MHC-I, pUS2 has been shown to target a range of cellular proteins for degradation, preventing their cell surface expression. Here we have identified a novel pUS2 target, ER-resident protein lectin mannose binding 2 like (LMAN2L). pUS2 expression was both necessary and sufficient for the downregulation of LMAN2L, which was dependent on the cellular E3 ligase TRC8. Given the hypothesized role of LMAN2L in the trafficking of glycoproteins, we employed proteomic plasma membrane profiling to measure LMAN2L-dependent changes at the cell surface. A known pUS2 target, integrin alpha-6 (ITGA6), was downregulated from the surface of LMAN2L-deficient cells, but not other integrins. Overall, these results suggest a novel strategy of pUS2-mediated protein degradation whereby pUS2 targets LMAN2L to impair trafficking of ITGA6. Given that pUS2 can directly target other integrins, we propose that this single viral protein may exhibit both direct and indirect mechanisms to downregulate key cell surface molecules.

## Introduction

Human cytomegalovirus (HCMV) is a ubiquitous herpesvirus with seroprevalence ranging from 45 to 100 %, depending on geographical location [1,3]. Following primary infection, HCMV establishes life-long latency which is normally controlled by a healthy immune system. However, HCMV infection and reactivation can cause substantial morbidity and mortality in immunocompromised individuals, and is the leading viral cause of congenital infection, affecting 1–5 % of pregnancies [4,6].

As with other *Herpesviridae*, HCMV has co-evolved with its human host and can exquisitely manipulate cellular processes to evade immune recognition and favour viral replication [7]. One way in which HCMV achieves this is by interfering with the ubiquitin proteasome system to promote degradation of cellular proteins. Our previous studies have shown that >900 cellular proteins are >3-fold downregulated during HCMV infection, and at least 133 are targeted for proteasomal or lysosomal degradation [8,9].

Targeted protein degradation is an important mechanism used by HCMV to prevent the cell surface expression of immune receptors and consequently evade immune recognition. The best described example is the degradation of the major histocompatibility complex class I (MHC-I) to inhibit antigen presentation to CD8^+^ T cells [10, 11]. HCMV pUS2 and pUS11 hijack the host’s endoplasmic reticulum (ER)-associated protein degradation (ERAD) pathway, through the recruitment of different E3 ligases, to ubiquitinate and translocate MHC-I molecules to the cytosol for degradation in the proteasome [12,19]. Antigen presentation is further impaired by the retention of MHC-I in the ER by pUS3 and pUS6 [20,21]. These strategies are also relevant for NK cell evasion, whereby multiple HCMV proteins degrade or sequester NK cell activating ligands that would otherwise stimulate NK cell cytotoxicity [22,25].

Targeting components of the host’s trafficking machinery is another way in which herpesviruses may indirectly prevent the surface expression or secretion of cellular proteins. Supporting this, one study showed that HCMV-encoded micro-RNAs (miRNAs) miR-UL112-1 and miR-US5-1 target members of the secretory pathway resulting in the impaired release of inflammatory cytokines IL-6 and TNF-α [26]. Similarly, we previously found that herpes simplex virus 1 (HSV-1) pUL56 degrades the cellular trafficking factor GOPC resulting in reduced expression of TLR2 at the plasma membrane [27].

To determine, in an unbiased fashion, novel mechanisms via which HCMV may modulate host trafficking, we compared our shortlist of proteins degraded during HCMV infection with a database of proteins involved in protein transport. Lectin mannose binding 2 like (LMAN2L) was the only protein in both databases for which the viral genetic locus required for degradation (US1-US11 region) is already known. Further analysis of individual genes within this locus showed that pUS2 is necessary for proteasomal degradation of LMAN2L, which it mediates by recruitment of cellular E3 ligase TRC8 (translocation in renal carcinoma, chromosome 8 gene). Proteomic plasma membrane profiling (PMP) identified that ITGA6 is an LMAN2L substrate, but other integrins are not. Overall, this study identified LMAN2L as a novel pUS2 target and suggests a novel, indirect viral mechanism of inhibition of cell surface protein expression.

## Methods

### Cell culture

Human foetal foreskin fibroblasts immortalized with human telomerase (HFFF-TERT), described previously [28], HFFF-TERTs overexpressing the coxsackie-adenovirus receptor (HFFF-CAR) and human embryonic kidney cells expressing SV40 large T antigen (HEK293T) were cultured in Dulbecco’s modified Eagle’s medium (DMEM; Sigma-Aldrich, 6429) supplemented with 10 % (v/v) FCS (Sigma-Aldrich, F7524) and incubated at 37 °C in a 5 % CO2 humidified environment. Cells were cultured in the absence of antibiotics for all experiments.

### Viruses

All viruses used in this study were derived from clinical strain Merlin (RCMV1111) and were generated by transfection with bacterial artificial chromosomes. HCMV strain Merlin is designated the reference HCMV sequence (RefSeq accession NC_006273.2) by the National Center for Biotechnology Information, and was originally sequenced after three passages in human fibroblast cells. RCMV1111 contains point mutations in two genes (RL13 and UL128) that enhance replication in fibroblasts [29,30]. ΔUS2 (RCMV1677, pAL1677), ΔUS3 (RCMV3226, pAL3226) and ΔUS11 (RCMV1579, pAL1579) single gene deletion mutants were generated on an RCMV1111 background as previously described [23,24, 30]. Recombinant adenoviruses (RAds) expressing individual ORFs were generated using the AdZ system and amplifying genes within the US1–US11 locus from Merlin DNA as previously described [31]. For all RAds, primers were designed to introduce a C-terminal V5 tag onto the insert.

### Viral infections

For HCMV infection, cells were seeded at 3×10^5^ cells per well in a six-well plate and incubated for 24 h. To enhance infection, cells were cultured in serum-free DMEM supplemented with dexamethasone (Sigma-Aldrich, D4902) at 4 µg ml^−1^ for 24 h prior to infection as previously described [32, 33, 10,34, 35]. Cells were infected at different multiplicities of infection (m.o.i.), as indicated in the text, in a total volume of 600 µl serum-free DMEM for 2 h at 37 °C on a rocking platform. The inoculum was removed and replaced with complete DMEM and cells were incubated for a further 24 h prior to harvesting.

HFFF-CAR cells were used for RAd experiments. HFFF-CAR cells were seeded into six-well plates at 3×10^5^ cells per well and incubated overnight. Cells were infected at an m.o.i. of 10 in 500 µl complete DMEM and incubated for 2 h at 37 °C on a rocking platform. The inoculum was removed and replaced with complete DMEM, and cells were incubated for 72 h before harvest for immunoblot or flow cytometry.

### siRNA knockdown

HFFF-TERTs were seeded in 10 cm dishes (2×10^6^ cells per dish) and incubated for approximately 24 h. Cells were transfected with a pool of LMAN2L small interfering RNA (siRNA) (Dharmacon, L-014658-02-0005) or non-targeting control siRNAs (Dharmacon, D-00181-10-20) at a final concentration of 20 nM, using liptofectamine RNAiMAX (Thermo Fisher Scientific, 56531). To improve LMAN2L knockdown efficiency, cells were passaged (1 in 2) 48 h after initial transfection and retransfected the following day. Cells were incubated for a further 72 h before sample preparation for PMP. siRNA target sequences are detailed in Table S1, available in the online version of this article.

### CRISPR/Cas9-mediated gene knockout

Low passage HFFF-TERTs were nucleofected with ribonucleoprotein (RNP) complexes containing recombinant Cas9 nuclease (IDT, 1081059) and guide RNAs (gRNA) against LMAN2L or a non-targeting negative control (Synthego), using the Nucleofector 2d Transfection Device (Lonza). An electroporation enhancer (IDT, 1075915) was added to cells prior to nucleofection to improve efficiency. Polyclonal (bulk knockout) samples were validated by immunoblot. Single guide RNA (sgRNA) sequences are detailed in Table S1.

### shRNA plasmid construction

Short hairpin RNA (shRNA) plasmids were constructed as previously described [8]. TRC8 shRNA oligonucleotides containing EcoRI and BamHI restriction site overhangs were adapted from Hse *et al*. [24]. Oligonucleotides were annealed and ligated into the pHR-SIREN vector (a gift from Prof. Paul Lehner, University of Cambridge) using T4 ligase (Thermo Scientific). The resultant shRNA construct was transformed into NEB 5-α competent *Escherichia coli* cells, followed by ampicillin selection. Non-targeting scrambled sequences was used as a control. All shRNA oligonucleotides are detailed in Table S1.

### Generation of stable cell lines by lentiviral transduction

Lentiviral particles were generated, as described previously [36], by transfection of 293Ts with expression vector and two lentiviral helper vectors (VSV-g, pCMV8.91) using TransIT-293 transfection reagent (Mirus, MIR 2700), according to he manufacturer’s instructions. Supernatants were collected 48 h post-transfection, and filtered onto HFFF-TERTs using a 0.22 µm filter. Transduced cells were placed in selection medium for 2 weeks with antibiotics.

### Immunoblotting

Radioimmunoprecipitation assay (RIPA) buffer (Cell Signaling Technology) containing 1× Roche complete Mini Protease Inhibitor Cocktail (Roche, 11836153001) was used to lyse cells. Samples were incubated for 30 min at 4 °C prior to centrifugation at 14 000 ***g*** for 10 min. Protein concentrations were measured using a BCA Protein Assay Kit (Thermo Fisher, 23227), according to the manufacturer’s instructions. Lysates were reduced by incubating samples with 6× Protein Loading Dye (X) at 95 °C for 5 min. Lysates containing a total of 50 µg of protein were separated by SDS-PAGE on a 4–20 % Mini-PROTEAN TGX Precast Protein Gel (Bio-Rad). Proteins were transferred to a PVDF membrane (0.45 µm pore) by wet transfer using a trans-blot system (Bio-Rad). Membranes were blocked with 5 % milk in TBS-T (TBS and 0.2 % v/v Tween) for at least 30 min at room temperature (RT). Membranes were probed overnight at 4 °C with the following antibodies: anti-GAPDH (1 : 10 000, R and D systems, MAB5718), anti-LMAN2L (1 : 100, Novus Biologicals, NBP1-84152) and anti-V5 (1 : 1000, Cell Signaling Technology, 132025). Membranes were washed three times with TBS-T and incubated for 1 h at RT with the following secondary antibodies: IRDye 680RD goat anti-mouse (LI-COR, 925-68070) and IRDye 800CW goat anti-rabbit (LI-COR, 925-32211). Results were acquired using a LI-COR Odyssey imager and images were processed using Image Studio Lite (LI-COR).

### RT-qPCR

Total RNA was extracted from TRC8 knockdown and control cell lines using the RNeasy Mini Kit (Qiagen, 74104) and contaminating DNA was removed using the TURBO DNA-free kit, according to the manufacturer’s instructions. The GoScript Reverse Transcription System was used to synthesize cDNA (Promega) and reverse transcription quantitative polymerase chain reaction (RT-qPCR) was performed using TaqMan Universal Universal Master Mix II with uracil-*N*-glycosylase (UNG) and TaqMan Gene Expression Assays with measuring TRC8 (Thermo Fisher, Hs00183680) and GAPDH (Thermo Fisher, 1807043) as an internal control. Results were acquired using the 7500 Fast and 7500 real-time PCR system (Applied Biosystems), using the following thermal cycling conditions: UNG incubation at 50 °C for 2 min, polymerase activation at 95 °C for 10 min, and 40 cycles of denaturation annealing/extension at 95 °C for 15 s and 60 °C for 1 min. Reactions were performed in triplicate and relative RNA levels were calculated using the 2^−ΔΔCT^ method [37].

### Flow cytometry

To validate expression of the V5-tagged transgene in cells infected with RAd vectors, cells were fixed with 4 % paraformaldehyde (PFA) for 10 min at RT and permeabilized with 100 % methanol at 4 °C for 10 min. Samples were incubated with TruStain FcX FC receptor blocking solution (diluted 1 : 10 in PBS/2 % FCS) for 10 min at 4 °C followed by incubation with anti-V5 (Cell Signaling Technology, 13202S) for 1 h at 4 °C, then by incubation with anti-rabbit IgG Alexa-Fluor 647 (Invitrogen, A31573) for 1 h at 4 °C. Results were acquired using a FACSCalibur Flow Cytometer (BD Biosciences) and analysed using FlowJo software (v.10.8.1, BD Biosciences).

### Plasma membrane profiling sample preparation

This method was adapted from our previous descriptions [9,38]. Cells seeded in 10 cm dishes were washed and incubated with an oxidation/biotinylation mix (1 mM sodium periodate, 100 µM aminooxy-biotin, 10 mM aniline in PBS pH 6.7) for 30 min at 4 °C in the dark. The reaction was quenched with addition of 1 mM glycerol solution, followed by two PBS washes. Cells were scraped in 1 ml lysis buffer (10 mM Tris/HCl, 1.6 % Triton, 150 mM NaCI, 1× cOmplete Mini EDTA-free Protease Inhibitor Cocktail and 5 mM iodoacetamide in H_2_O) and were incubated for 30 min on ice. To pellet nuclei and cell debris, samples were centrifuged for 5 min (13 000 ***g***, 4 °C) three times, transferring to new tubes after every spin. Clarified lysates were incubated with highaffinity streptavidin agarose beads for 75 min at 4 °C followed by sequential washes with lysis buffer, 0.5 % SDS in PBS and urea solution (6 M urea in 0.1 M Tris/HCl). Bead-bound proteins were reduced with 100 mM DTT in PBS/0.5 % SDS, and were alkylated using 50 mM iodoacetamide (IAA) in urea solution. Samples were digested with modified sequencing-grade trypsin in 35 µl 200 mM HEPES pH 8.5 for 3 h at 37 °C.

### Labelling peptides with tandem mass tags

Each 0.5 mg vial of TMTproTM 16plex reagents (Thermo) was dissolved in 45 µl anhydrous AcN (Acros Organics). To ensure an overall AcN concentration of 28–30 % (v/v), 5 µl of AcN was added and samples were labelled with 10 µl TMT reagent in a total volume of 50 µl, as described previously [27]. Samples were incubated for 1 h at RT. For initial analysis, 10 % of each sample was combined. This was to ensure similar peptide loading across each TMT channel, and that TMT-labelling was >95 % incorporated, prior to combination for fractionation. Correction factors, calculated according to the relative peptide levels, were used to adjust the volume of each sample when combining the remaining samples. Combined samples were quenched with hydroxylamine (Thermo) at a final concentration of 0.5 % (v/v). Samples were desalted using a StageTip prior to analysis. Samples were labelled as detailed in Table S1.

### Offline high pH reversed-phase chromatography (HpRp) fractionation

Prior to fractionation, combined samples were desalted using Sep-Paks (Waters) and vacuum-centrifuged to near dryness. Fractionation was performed, as previously described [8], using an Ultimate 3000 rapid separation liquid chromatography (RSLC) nano ultra-high-performance chromatography (UHPLC) system equipped with a 2.1 mm internal diameter ×25 cm long, 1.7 µm particle Kinetix Evo C18 column (Phenomenex). The mobile phase was as follows: 3 % AcN (A), 100 % AcN (B) and 200 mM ammonium formate pH 10 (C). Mobile phase C was maintained at 10 %, whereas A and B were altered over the course of the gradient. The gradient elution consisted of 0–19 % B over 10 min, 19–34 % B over 14.25 min and 34–50 % B over 8.75 min, followed by a 10 min wash with 90 % B. The flow rate was set to 200 µl min^−1^ for the initial 5 min whilst the sample was loaded, and then was increased to 400 µl min^−1^ for the rest of the protocol. Fractions were collected every 15 s in 96-well plates and UV absorbance at 280 nm was measured over the course of the fractionation, generating a chromatogram that was used to identify peptide-rich fractions for combination. Adjacent columns of wells were combined into six distinct fractions for analysis.

### LC-MS3

TMT-labelled samples were analysed using an Orbitrap Fusion Lumos (Thermo Fisher Scientific) coupled to an UltiMate RSLC3000 UPLC, as detailed by Soh *et al*. [27]. Peptides were separated using a 300 µm internal diameter ×5 mm Acclaim PepMap µ-Precolumn (Thermo). Loading solvent was 0.1 % FA, and analytical solvent A: 0.1 % FA and B: 80 % MeCN+0.1 % FA. All separations were carried out at 55 °C. Samples were loaded at 5 µl min^–1^ for 5 min in loading solvent before beginning the analytical gradient. The following gradient was used: 3–7 % B over 3 min, 7–37 % B over 116 min, followed by a 4 min wash at 95 % B and equilibration at 3 % B for 15 min. Each analysis used a MultiNotch MS3-based TMT method [39]. The following settings were used: MS1: 380–1500 Th, 120 000 resolution, 2×10^5^ automatic gain control (AGC) target, 50 ms maximum injection time; MS2: quadrupole isolation at an isolation width of *m*/*z* 0.7, CID fragmentation [normalized collision energy (NCE) 35; with ion trap scanning in turbo mode from *m*/*z* 120, 1.5×10^4^ AGC target, 120 ms maximum injection time and MS3: in Synchronous Precursor Selection mode, the top six MS2 ions were selected for HCD fragmentation (NCE 65) and scanned in the Orbitrap at 60 000 resolution with an AGC target of 1×10^5^ and a maximum accumulation time of 150 ms. Ions were not accumulated for all parallelizable time. The entire MS/MS/MS cycle had a target time of 3 s. Dynamic exclusion was set to ±10 p.p.m. for 70 s. MS2 fragmentation was trigged on precursors 5×10^3^ counts and above.

### Data analysis

The human UniProt database (26 January 2017) was combined with a database of common contaminants for the processing of all raw data. The database was concatenated with a reverse database composed of all protein sequences in reverse which served as ‘decoy’ sequences to estimate a false discovery rate (FDR). Searches were performed using a 20 p.p.m. precursor ion tolerance. Product ion tolerance was set to 0.03 Th. Oxidation of methionine residues (15.99492 Da) was set as a variable modification. FDR for peptide spectral matches was set to 0.02 (2 %), using linear discrimination analysis to score spectral matches, and further filtered to a protein-level FDR of 1 %. Protein assembly was guided using the principles of parsimony, where peptides were matched to the smallest set of proteins that account for all peptides measured.

Raw files were processed, as previously described [8], using a Sequest-based software pipeline, MassPike, licensed by Harvard Medical School and accessed through a collaborative arrangement with Professor Steven Gygi’s laboratory. MS spectra were converted to mzXML using an extractor built upon Thermo Fisher’s RAW File Reader library (version 4.0.26). In this extractor, the standard mzXML format has been augmented with additional custom fields that are specific to ion trap and Orbitrap MS and essential for TMT quantification. These additional fields include ion injection times for each scan, Fourier transform-derived baseline and noise values calculated for every Orbitrap scan, isolation widths for each scan type, scan event numbers, and elapsed scan times. Static modifications on lysine residues and peptide N termini (229.162932 Da) and carbamidomethylation of cysteine residues (57.02146 Da) were included for processing TMT-labelled samples. Proteins were quantified by summing the TMT reporter signal from all peptide spectral matches for that parent protein. Briefly, a 0.003 Th window around the theoretical *m*/*z* of each reporter ion (126, 127n, 127c, 128n, 128cc, 129, 129c, 130n, 130c, 131n, 131c, 132n, 132c, 133n, 133c, 134n) was scanned for ions, and the maximum intensity nearest to the theoretical *m*/*z* was used. The primary determinant of quantification quality is the number of TMT reporter ions detected in each MS3 spectrum, which is directly proportional to the signal-to-noise (S:N) ratio observed for each ion. Conservatively, every individual peptide used for quantification was required to contribute sufficient TMT reporter ions so that each on its own could be expected to provide a representative picture of relative protein abundance, requiring a summed S:N of at least 250 [39]. An isolation specificity filter with a cutoff of 50 % was additionally employed to minimize peptide co-isolation. Peptides that satisfied this criterion were summed, giving an overall S:N value for that protein. These values were exported for further analysis in Excel. First, reverse hits and contaminants were removed, and values were normalized by summing S:N values for every protein in each channel and calculating normalization factors (relative to the first channel) which were used to scale all other channels.

Gene Ontology Cellular Compartment (GOCC) terms were assigned to proteins, and plasma membrane proteins were classified as those assigned with any of the following terms: plasma membrane (PM), cell surface but not PM (CS), extracellular but not PM/CS (XC) or with a short GO term (ShG), referring to proteins annotated by GO as integral to the membrane, but with no subcellular assignment and a short four- or five-part GO cellular compartment term as previously described [38]. Fold changes (LMAN2L knockdown/control) were averaged across siRNA replicates.

A coefficient of variation (CV) was calculated by dividing the standard deviation by the average fold change across siRNA and bulk CRISPR samples, and multiplying by 100. Entries with CV >30 were excluded from further analysis.

Significance A was used to estimate *P*-values and the method of Benjamini–Hochberg was used to adjust *P*-values for multiple hypothesis testing in Perseus version 1.6.0.3 [40,41].

All normalized data, with reverse hits and contaminants removed, are included in Table S2.

The *P*-values for Figure 2c were calculated using a Student’s two-sample, unpaired t-test in R version 4.2.2 (R Core Team 2022).

### Data availability

The MS proteomics data have been deposited to the ProteomeXchange Consortium (http://www.proteomexchange.org/) via the PRIDE partner repository [42] with the dataset identifier PXD050878. All materials described in this paper and any further details of protocols employed can be obtained on request from the corresponding author by email.

## Results

### LMAN2L is a novel HCMV US2 target

**Fig. 1.**
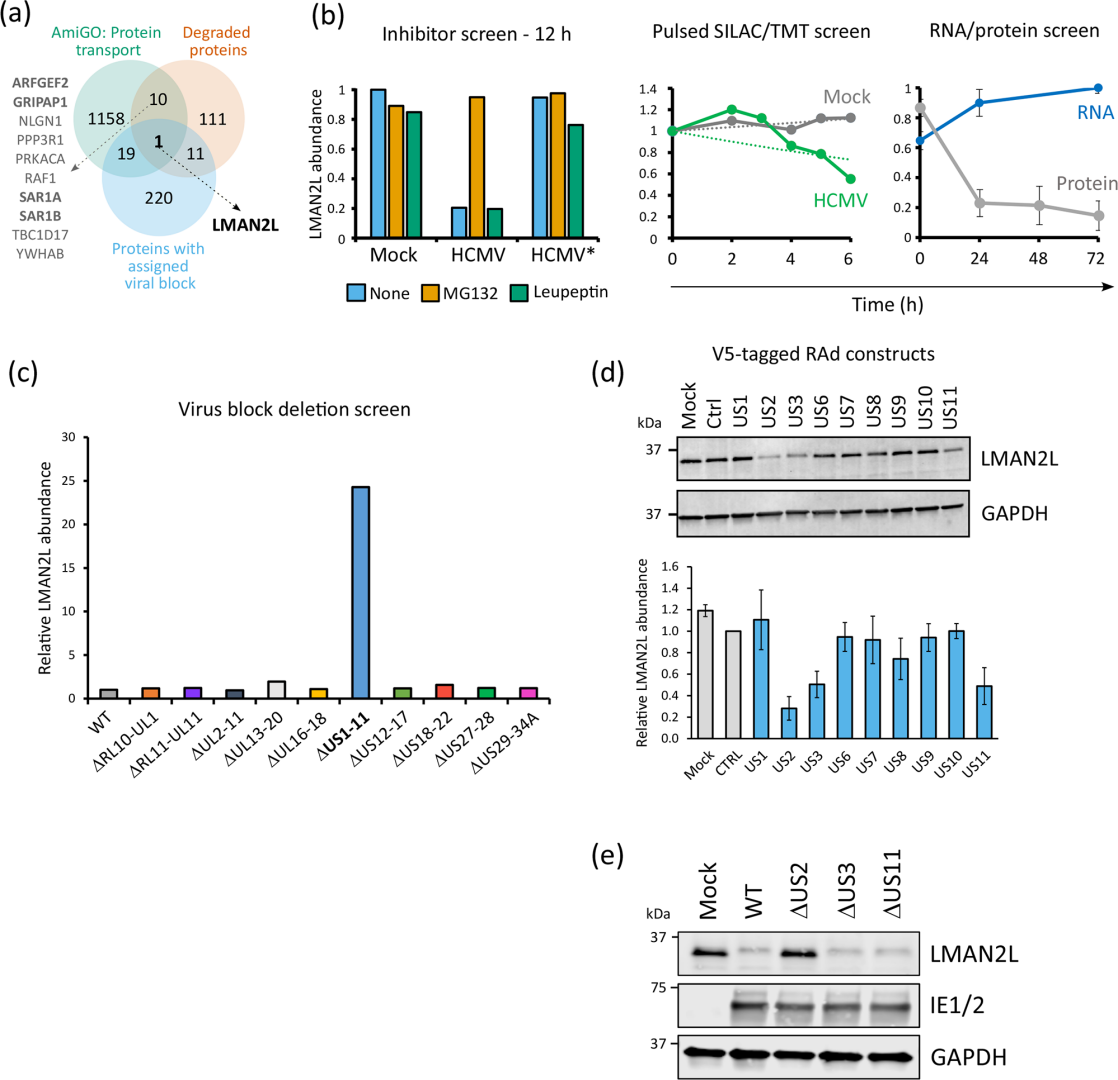
US2 degrades LMAN2L via the proteasome. (a) Venn diagram showing the overlap between shortlists of proteins: (i) degraded early during HCMV infection [8]; (ii) significantly rescued upon infection with an HCMV gene-block deletion mutant compared to wild-type infection [55]; and (iii) assigned the gene ontology term ‘protein transport’ (GO:0015031) according to the AmiGO database [44, 43]. The following two panels (b, c) are based on data from our prior publication [55], and are the only previously published data in this paper. (b) Three orthogonal protein degradation screens showing that LMAN2L is degraded with medium confidence during early HCMV infection (from [55]). Left panel: LMAN2L was rescued from degradation by the proteasome inhibitor MG132 in HCMV-infected cells, but not during mock infection, infection with UV-irradiated HCMV (HCMV*) or inhibition with the lysosomal protease inhibitor leupeptin (see also figure 1 from [55]). Middle panel: increased rate of LMAN2L degradation from 4 h of HCMV compared to mock infection. Cells were pre-labelled with medium SILAC amino acids prior to infection, then switched to heavy amino acids at the point of infection. Quantification of medium-labelled proteins over time using tandem mass tag (TMT)-based multiplexing facilitated quantification of protein degradation rates (see also figure 2 from [55]). Right panel: relative abundance of LMAN2L protein and transcript over 72 h of infection. (c) A proteomic screen of HCMV block deletion mutants determined that the US1–US11 gene block is required to downregulate LMAN2L [55]. Abundance is shown relative to WT HCMV. A value of 1 indicates no change. (d) Immunoblot showing expression of endogenous LMAN2L in HFFF-CAR infected with RAd expressing V5-tagged US1–US11 genes (m.o.i. 10, 72 h). LMAN2L abundance was normalized to GAPDH and is shown relative to the control RAd given a value of 1. Error bars represent the range (*n*=2). Validation of transgene expression is shown in Fig. S1. (e) Immunoblot of lysates from HFFF-TERTs infected with strain Merlin HCMV, and ΔUS2, ΔUS3 or ΔUS11 recombinants (m.o.i. 5, 24 h), or mock infected. Immunoblot shown is representative of two independent experiments.

To investigate alternative novel mechanisms via which HCMV may indirectly alter protein expression at the cell surface, we compared a shortlist of proteins degraded early during HCMV infection that we previously defined [8], with a database of proteins involved in protein transport acquired from AmiGO using the protein transport accession GO:0015031 [43,45] (Table S3). Of 133 degraded proteins identified in the ‘high’ or ‘medium confidence’ shortlist, 11 also had a role in protein transport (Fig. 1a). These included ADP-Ribosylation Factor Guanine Nucleotide-Exchange Factor 2 (ARFGEF2), important for trans-Golgi network integrity and trafficking [46,48]; GRIP1 Associated Protein 1 (GRIPAP1), involved in the regulation of endosomal recycling [49]; Secretion Associated Ras Related GTPase 1 paralogues (SAR1A and SAR1B), components of coat protein complex II (COPII) that facilitate transport of proteins from the ER [50,51]; and Lectin mannose binding 2-like (LMAN2L), with hypothesized roles in the early secretory pathway [52,53]. ARFGEF2 and GRIPAP1 have been implicated in protein expression at the plasma membrane. Inhibition of ARFGEF2 impairs E-cadherin and β-catenin trafficking to the cell surface [47]. GRIPAP1 mutations hinder endosomal recycling of synaptic AMPA-type glutamate receptors [49]. However, the potential role of these proteins with regard to HCMV-mediated changes at the cell surface is unknown.

**Fig. 2.**
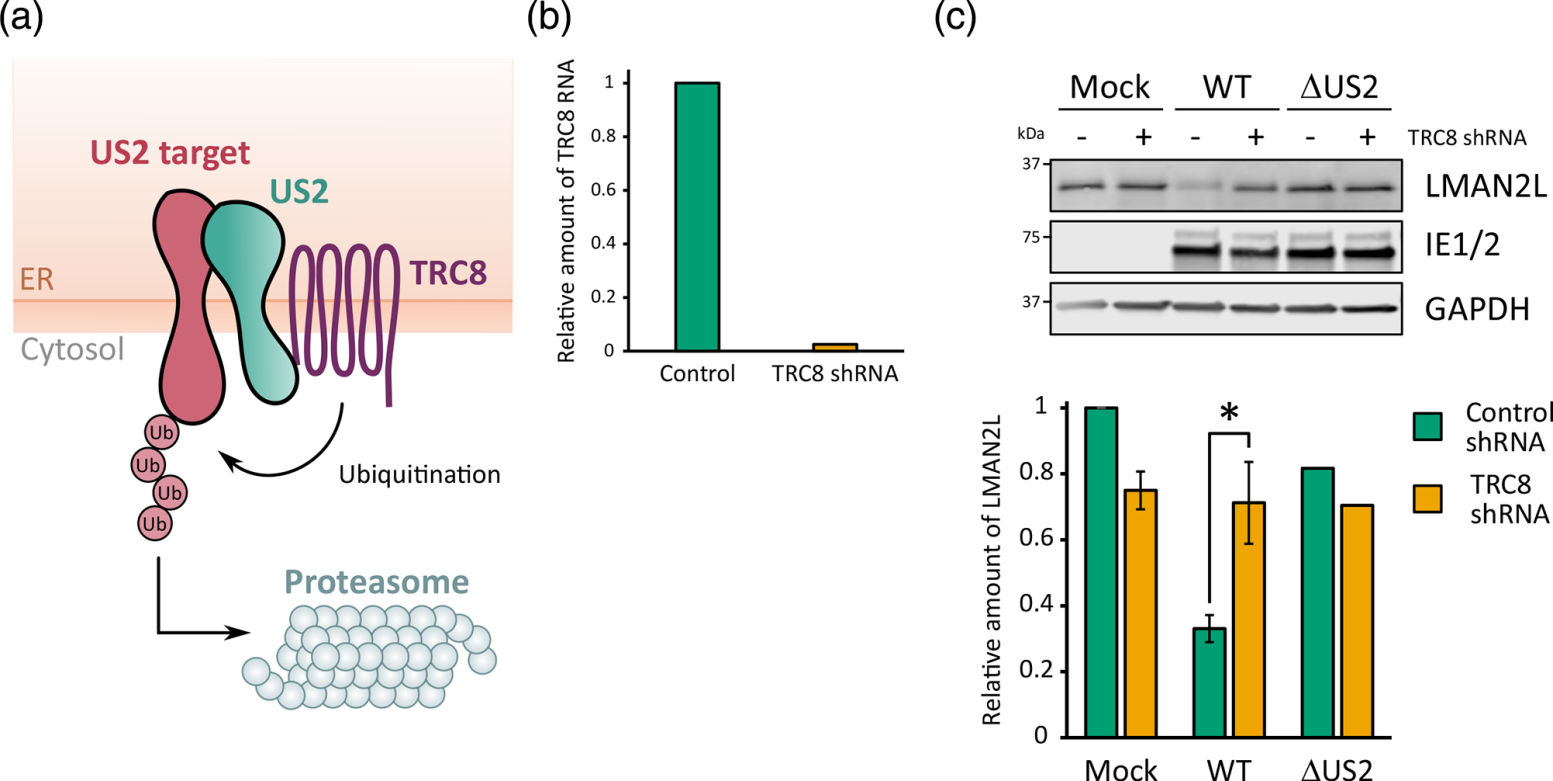
Cellular E3 ligase TRC8 is necessary for LMAN2L degradation. (**a**) Schematic of the mechanism of US2 and TRC8-mediated degradation of membrane proteins, adapted from Hsu *et al*. [24]. (**b**) Confirmation of TRC8 knockdown. HFFF-TERTs were transduced with TRC8 or scrambled control shRNA plasmids and knockdown efficiency was quantified by RT-qPCR. Relative RNA levels were calculated using the 2^−ΔΔCT^ method [37]. (**c**) LMAN2L expression in HCMV-infected TRC8 shRNA knockdown cells. Control (-) and TRC8 shRNA (+) cell lines were infected with strain Merlin WT HCMV or a ΔUS2 strain Merlin recombinant for 24 h (m.o.i. 5), and LMAN2L expression was measured by immunoblot. The LMAN2L signal was normalized to GAPDH and is shown relative to mock control samples. The average relative abundance is shown across three biological replicates and error bars represent the standard error of the mean, except for samples infected with ΔUS2 where there were no biological replicates. *P*-values were calculated using a two-sample unpaired t-test. *P<0.05.

We previously employed a panel of viral mutants to predict blocks of HCMV genes that target human proteins [8]. Of the 11 degraded proteins with roles in protein transport, LMAN2L was the only factor ‘rescued’ from degradation upon infection with a block deletion mutant compared to wild-type infection (Fig. 1a), highlighting its tractability for further investigation. We have also previously demonstrated that LMAN2L was downregulated as early as 4 h post-infection and was rescued upon addition of proteasome inhibitor MG132, but not lysosome inhibitor leupeptin, and that its transcript levels were not downregulated [8] (Fig. 1b). Deletion of the HCMV US1-US11 locus resulted in a >20-fold increase in LMAN2L protein abundance relative to wild-type (WT) HCMV-infected cells [8] (Fig. 1c).

LMAN2L is an ER-resident L-type lectin that binds glycoproteins and is hypothesized to assist their trafficking to the Golgi apparatus [52,55]. LMAN2L selectively interacts with deglucosylated high-mannose-type N-glycans, which is characteristic of mature glycoproteins that have undergone correct protein folding [52,56,58]. LMAN2L may assist receptor-mediated transport of mature glycoproteins out of the ER in COPII-coated vesicles [52].

To determine which of the US1–US11 gene(s) target LMAN2L for degradation, HFFF-TERTs stably expressing the human coxsackievirus and adenovirus receptor (HFFF-CAR) were transduced with RAds each expressing one or other of the genes in the US1–US11 locus. Expression was validated by immunoblot or intracellular flow cytometry for all but RAd-US9 (Fig. S1). Expression of pUS2, pUS3 and pUS11 downregulated LMAN2L compared to mock infection and control RAd (Fig. 1d).

To assess whether pUS2, pUS3 or pUS11 are necessary for HCMV-mediated LMAN2L downregulation, cells were infected with single gene deletion mutants or with WT HCMV. Only infection with ΔUS2 rescued LMAN2L expression to mock levels, whereas LMAN2L was still downregulated in the absence of pUS3 and pUS11 (Fig. 1e). While viral fitness was not formally assessed, ΔUS2 recombinant virus grew normally in cell culture and to similar titres as WT virus (data not shown). Furthermore, IE1/2 protein expression was similar between samples (Fig. 1e), suggesting that there was not a defect in viral gene expression across WT and single gene mutant virus infection. Overall, this suggests that pUS2 alone is necessary for LMAN2L degradation during infection and that this phenotype is unlikely to be a result of impaired viral replication.

### HCMV-induced LMAN2L degradation is dependent on TRC8

pUS2 is expressed early during infection, consistent with the kinetics of LMAN2L degradation, and localizes to the ER, where it highjacks the ERAD pathway to target cellular proteins for proteasomal degradation in the cytosol [11,16]. The cellular E3 ligase TRC8 is required for degradation of all pUS2 substrates so far examined, such as MHC-I, NK cell activating receptor CD112, IL-12 receptor and a subset of integrin α subunits [16,24]. pUS2 binds its substrate and TRC8 via its luminal domain and cytoplasmic tail, respectively [13,24, 59, 60], which promotes substrate ubiquitination, ER-to-cytosol dislocation and subsequent proteasomal degradation (Fig. 2a) [16,61]. To determine whether LMAN2L downregulation is similarly TRC8-dependent, stable shTRC8 knockdown and control cell lines were generated and validated by RT-qPCR, which showed a 40-fold decrease in TRC8 expression in knockdown compared to control cells (Fig. 2b). Depletion of TRC8 rescued LMAN2L expression in WT HCMV-infected samples (Fig. 2c), suggesting that this ligase is required to degrade LMAN2L, probably via pUS2-mediated recruitment.

### Surface ITGA6 is reduced on LMAN2L-deficient cells in the absence of infection

**Fig. 3.**
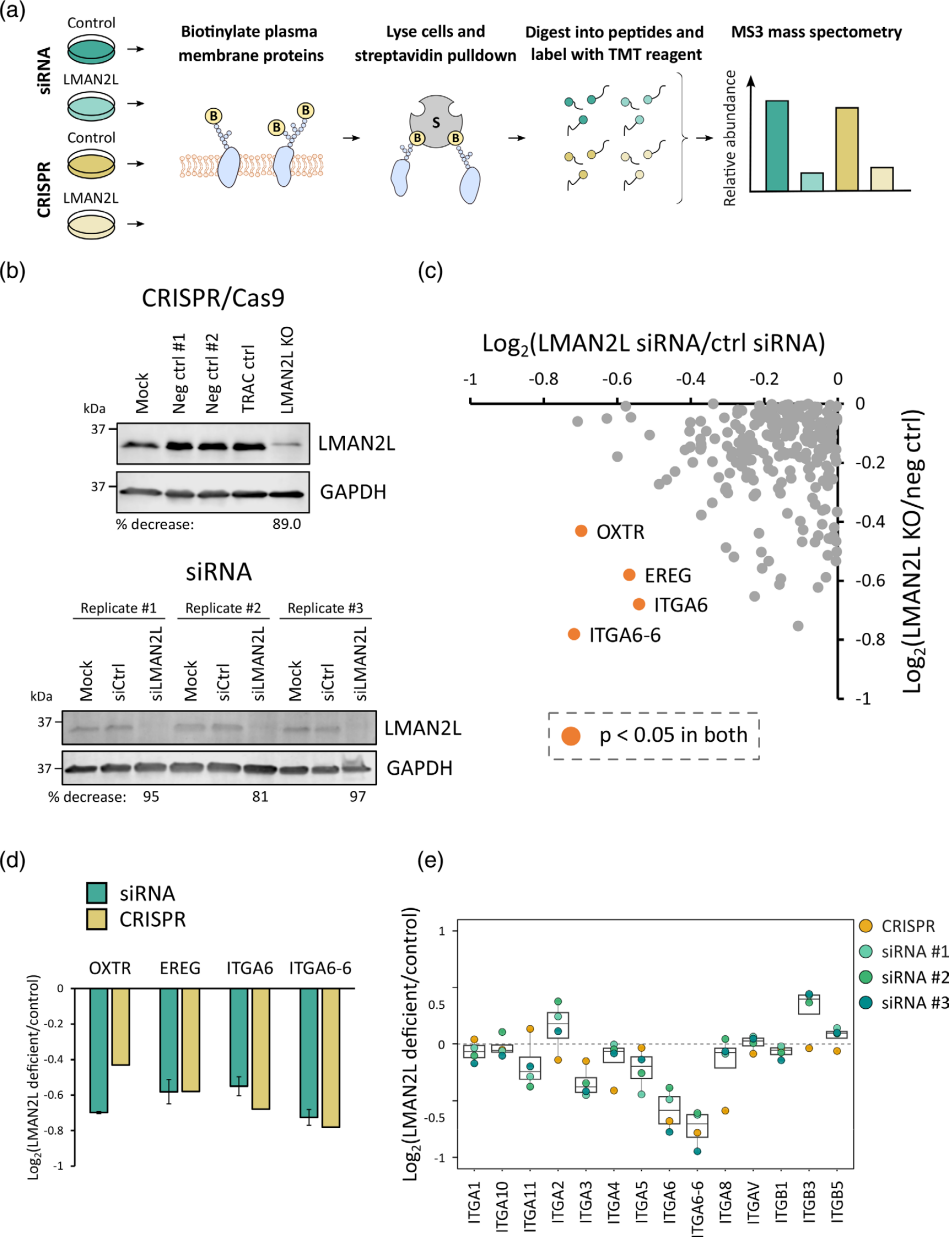
LMAN2L-dependent expression of proteins at the plasma membrane. (**a**) Schematic of the PMP workflow. (**b**) Immunoblot validation of LMAN2L knockdown/knockout in bulk CRISPR KO cells (top) and after siRNA treatment (bottom). Percentage decrease was calculated relative to the corresponding negative controls (ctrl). (**c**) Scatterplot comparing the average fold change from siRNA (*n*=3) and CRISPR samples (*n*=1). A coefficient of variation (CV) was calculated by dividing the standard deviation by the average fold change across siRNA and bulk CRISPR samples and multiplying by 100. Entries with a CV>30 were excluded from further analysis. Significance A was used to calculate *P*-values, which were corrected for multiple testing using the Benjamini–Hochberg method [40,41]. Proteins that were significantly downregulated (*P*<0.05) in both LMAN2L siRNA and LMAN2L bulk KO are labelled and highlighted in orange. (**d**) Fold change of proteins significantly downregulated in both LMAN2L siRNA knockdown and bulk LMAN2L KO cells compared to the corresponding control. Error bars represent the standard error of the mean. (**e**) Fold change of all integrin molecules quantified in the proteomic screen. The bottom and top of the box show the first and third quartile, respectively, and the median is shown as the line inside the box. Individual fold changes for the replicates are shown as data points.

Whilst the function of LMAN2L is not well characterized, its ER localization and affinity for deglucosylated glycans are suggestive of a role in trafficking of mature glycoproteins from the ER to the Golgi [52,62, 63]. Although most plasma membrane proteins encode ER export motifs that directly interact with COPII coat proteins, some require additional assistance from cargo receptors that provide another signal for ER export [64,65]. We hypothesized that LMAN2L may direct the receptor-mediated ER export of certain cellular glycoproteins and that its degradation by HCMV may indirectly prevent the expression of LMAN2L-dependent cargo at the plasma membrane. To identify LMAN2L-dependent plasma membrane proteins, we employed PMP, our method for isolation of highly purified plasma membrane proteins for proteomic analysis. TMT reagents were used to multiplex all conditions (Fig. 3a). As pUS2 is known to directly target proteins for degradation, thereby inhibiting their cell surface expression, a ΔUS2 virus could not be used to identify LMAN2L-dependent substrates. Instead, two orthogonal methods of gene knockdown were employed: siRNA knockdown and bulk CRISPR knockout (KO) (Fig. 3b). We aimed to identify proteins with reduced abundance in LMAN2L knockdown and bulk KO samples compared to controls as an indicator of their dependence on LMAN2L for cell surface trafficking.

Overall, 948 proteins were quantified with gene ontology annotations of ‘plasma membrane’, ‘cell surface’, ‘extracellular’ or with a ‘short GO’ term, which refers to a subset of proteins annotated by GO as integral to the membrane but with no subcellular assignment and a four- or five-part GO cellular compartment term [38]. Data from this proteomic experiment are shown in Table S2. Here, the worksheet ‘Plots’ is interactive, enabling generation of graphs of protein expression of any of the proteins quantified. Four proteins were found to be significantly downregulated in both siRNA and CRISPR samples: oxytocin receptor (OXTR), proepiregulin (EREG), integrin alpha-6 (ITGA6) and isoform alpha-6X1X2A of ITGA6 (ITGA6-6) (Fig. 3c, d). Although two ITGA6 isoforms were quantified, the lack and unique peptides and the redundancy across isoforms make it challenging to accurately characterize isoforms from these data. Considering this, no isoform specific conclusions can be drawn.

Of 13 α or β integrins quantified, only ITGA6 was robustly downregulated in both siRNA and CRISPR samples (Fig. 3e). Other integrins were modulated in either set of samples but not both. Lack of consistent regulation was considered to exclude a significant effect. The downregulation of ITGA6 and ITGA6-6, although modest, was particularly interesting given that pUS2 is known to target a subset of integrins, including ITGA6, for degradation in a TRC8-dependent manner [24]. As knockdown of LMAN2L did not result in global downregulation of integrins at the cell surface, we speculate that this is a complementary mechanism of pUS2-mediated ITGA6 downregulation rather than the foundational mechanism underlying the degradation of multiple integrin α subunits by pUS2. OXTR and EREG are not known to be degraded by pUS2, but may be secondary targets resulting from the degradation of LMAN2L. Overall, degradation of LMAN2L may therefore represent a complementary, indirect mechanism via which pUS2 can alter the surface expression of ITGA6, and other potential targets.

## Discussion

The long-term co-evolution of HCMV with its human host has resulted in the development of diverse strategies to thwart antiviral defences and subvert cellular processes in favour of viral replication. HCMV manipulates the host’s degradation machinery to degrade antiviral restriction factors, alter the expression of immune receptors and regulate viral protein abundance [66]. In the ER, viral proteins, including pUS2 and pUS11, hijack the ERAD pathway to target key immunomodulatory proteins from the ER to the cytosol for proteasome-mediated degradation [19, 14,61, 12, 16], 18].

Here, we have identified lectin-mannose binding protein LMAN2L as a novel target of pUS2. Like other pUS2 targets [16,24], depletion of TRC8 rescued LMAN2L expression during infection. pUS2 interacts with TRC8 and situates its substrates for ubiquitination [16,24]. This, and the recruitment of other components of the ERAD machinery, results in the translocation and proteasomal degradation of substrate into the cytosol [19, 13,67, 68]. pUS2 expression in the absence of infection is sufficient for the downregulation of its targets, which was also true for LMAN2L where infection with recombinant adenovirus expressing US2 reduced LMAN2L expression (Fig. 1d).

Unlike all previously characterized pUS2 targets that are expressed from the ER through the secretory pathway to the plasma membrane, LMAN2L localizes solely to the ER. Immunofluorescence studies suggest it is retained in the ER and does not cycle in the early secretory pathway [53,55]. This is supported further by the identification of an RKR ER retention motif at its cytoplasmic domain and the optimal binding of LMAN2L with glycan substrates at a neutral pH, corresponding to the luminal pH of the ER [52,54]. One study found occasional overlap in expression between Golgi marker mannosidase II and overexpressed myc-tagged LMAN2L, suggesting it may cycle to the Golgi [53]. However, this may be an artefact of overexpression and/or protein tagging. Another study showed that treatment with brefeldin A, a drug known to redistribute rapidly cycling proteins to the ER–Golgi intermediate compartment (ERGIC), did not alter the localization of LMAN2L from the ER [54], supporting the characterization of LMAN2L as a non-cycling ER protein.

The folding and subsequent transport of glycoproteins out of the ER is regulated by quality control sensors that recognize different glycan intermediates and protein signals, distinguishing mature and immature cargo. Misfolded glycoproteins are selectively retained in the ER through an affinity with UDP-Glc:glycoprotein glucosyltransferase 1 (UGGT1) which reglycosylates high-mannose glycans and promotes the reassociation of glycoproteins with molecular chaperones, calnexin and calreticulin, that assist folding [69,70]. The glycans of terminally misfolded glycoproteins are successively modified by mannosidases, ultimately preventing their reassociation with UGGT1 and instead promoting recognition by components of the ERAD machinery [57,71,74]. By contrast, correctly folded glycoproteins are recruited to ER exit sites through direct interaction with COPII coat proteins or with intermediate cargo receptors, and are not extensively demannosylated [63,75]. LMAN2L selectively interacts with deglucosylated high-mannose-type N-glycans, characteristic of mature glycoproteins. The affinity for interaction is reduced upon glucosylation and removal of mannose residues, further indicating a selectivity for correctly folded glycoproteins [52,58]. Kamiya *et al*. [52] proposed that LMAN2L binds mature deglucosylated glycoproteins as they leave the calnexin/calreticulin/UGGT1 folding cycle and may act as an intermediate cargo receptor. Since LMAN2L is an ER-resident protein and is unlikely to cycle to the Golgi, it may cooperate with a cycling cargo receptor to enable anterograde transport of certain substrates [52].

To assess whether LMAN2L expression influences transport of proteins to the cell surface, we used PMP to compare control and LMAN2L-deficient cells. A particular advantage of the orthogonal siRNA- and CRISPR-based approach employed was the high-confidence identification of LMAN2L substrates. These included both integrin alpha-6 (ITGA6) and a non-canonical isoform of ITGA6 (ITGA6-6) (Fig. 3c-e), further increasing confidence that ITGA6 is a bona fide LMAN2L substrate. This was of particular interest as ITGA6 has previously been identified as a target of pUS2 [24]. This study showed that pUS2 degrades a subset of integrin α subunits in a TRC8-dependent manner, and co-precipitates with ITGA4. It was therefore suggested that integrins are direct targets of pUS2 [24]. While not assessed for ITGA6, pUS2 was found to interact with ITGA4 and has previously been shown to interact directly with other substrates in the absence of other cellular factors [24,59, 60]. Although ITGA6 degradation has not been assessed in the absence of LMAN2L, we speculate that LMAN2L degradation is an additional, indirect method to downregulate surface ITGA6 which complements direct pUS2-mediated degradation. No other integrin α subunits were downregulated on LMAN2L-deficient cells, despite being known pUS2 targets, further supporting the hypothesis that the degradation of LMAN2L is not a core mechanism fundamental to pUS2 activity.

The modest but consistent downregulation of surface ITGA6 across screens of orthogonally derived knockdowns may reflect a delay in trafficking rather than an inability to reach the plasma membrane in the absence of LMAN2L. Considering its high selectivity for deglucosylated high-mannose glycans, indicative of correctly folded glycoproteins, LMAN2L may instead act as a quality control sensor for protein folding rather than a key trafficking factor. Therefore, LMAN2L degradation may temporarily retain substrates in the ER rather than preventing trafficking to the plasma membrane. This could enrich the ER with pUS2-specific substrates and improve efficiency of direct, pUS2-mediated degradation of this protein. A similar mechanism has been proposed for the downregulation of two pUS2 targets, MHC-I and NK cell activating ligand CD112 [10,24]. Surface expression of CD112 is downregulated by pUS2 and pUL141, both of which can achieve this in isolation [24,25]. However, co-expression of US2 and UL141 results in synergistic downregulation that is greater than the sum of their separate effects. UL141 does not actively degrade its targets but instead retains them in the ER to prevent cell surface expression [25,76]. This retention, and the probable interaction between pUS2 and pUL141, enhances pUS2-dependent degradation of the mutual target [24]. A similar mechanism has been suggested for pUS3 in the downregulation of MHC-I, whereby pUS3 retains MHC-I in the ER and enhances pUS2- and pUS11-dependent degradation [10,21].

Integrin molecules exist as heterodimers at the cell surface, comprising α and β subunits which function together as receptors for the extracellular matrix and activate intracellular signalling pathways that regulate cell adhesion and migration. ITGA6 associates with ITGB1 and ITGB4 to form α6β1 and α6β4 integrin complexes that bind laminins [77,79]. Interestingly, α6β1 complexes are important for entry of HCMV into fibroblasts, and infection results in β1-integrin-specific signalling, demonstrating a proviral role during the initial stages of replication [80]. In THP-1 cells, ITGA6 is induced at 96 h post-infection in the absence of pUS2/TRC8-mediated degradation [24], suggesting an antiviral role in this cellular context and/or stage of infection.

Two additional proteins were shortlisted from the PMP experiment in addition to ITGA6, namely EREG and OXTR. EREG is expressed at the plasma membrane and is cleaved by cell surface proteases, releasing the soluble ectodomain that binds epidermal growth factor receptor (EGFR) and activates a signalling cascade that stimulates cell proliferation [81,83]. OXTR is a receptor for the peptide hormone oxytocin that is primarily known for its role in regulating processes related to reproduction, but has been associated with cell proliferation and tumorigenesis [84,85]. Although these proteins are functionally distinct and have different physiological roles, they operate in pathways that are somewhat interconnected and lead to related cellular responses. This is especially true for integrin and EGFR signalling pathways which are known to cross-talk [86,89]. Interestingly, high integrin α6β4 in pancreatic cancer cells is associated with transcriptional upregulation of EGFR ligands, including EREG, which activates EGFR signalling and enhances cell motility and migration [90]. Furthermore, pUS2-mediated downregulation of integrins is associated with reduced cell adhesion and motility. Given the roles of proteins identified as potential LMAN2L cargo, it would be interesting to determine whether LMAN2L degradation impacts cell migration during infection.

Two proteins within the same family as LMAN2L, namely ERGIC-53 and LMAN2, have been identified as cargo receptors involved in the trafficking of certain secretory proteins [91,93]. Correspondingly, one study showed that knockdown of LMAN2L resulted in the delayed secretion of two unknown glycoproteins [53]. Considering this, and the identification of secretory protein EREG in the PMP shortlist, LMAN2L degradation may have an additional effect on secretory proteins. Therefore, future studies should also focus on measuring the global impact of LMAN2L downregulation on protein secretion in the context of infection.

Modulation of cell surface protein expression is an important strategy employed by HCMV to evade immune recognition [22,23, 76, 94, 95]. Understanding these mechanisms can inform the development of anti-HCMV therapies to overcome viral antagonism of host immunity. These results identify an additional pUS2 target and highlight a complementary strategy via which pUS2 interferes with cellular quality control and trafficking pathways, through the degradation of LMAN2L, to prevent cell surface expression of one of its targets.

## supplementary material

10.1099/jgv.0.001980 Uncited Supplementary Material 1.

## Abbreviations

ER: endoplasmic reticulum
ERAD: endoplasmic reticulum-associated degradation
HCMV: human cytomegalovirus
HFFF-TERT: human foetal foreskin fibroblasts immortalized with human telomerase
ITGA6: integrin alpha-6
KO: knockout
LMAN2L: lectin mannose binding 2 like
MHC-I: major histocompatibility complex class I
m.o.i.: multiplicity of infection
PM: plasma membrane
PMP: plasma membrane profiling
TMT: tandem mass tag
TRC8: translocation in renal carcinoma, chromosome 8 gene
WT: wild-type
